# Strong inverse kinetic isotope effect observed in ammonia charge exchange reactions

**DOI:** 10.1038/s41467-019-13976-8

**Published:** 2020-01-10

**Authors:** L. S. Petralia, A. Tsikritea, J. Loreau, T. P. Softley, B. R. Heazlewood

**Affiliations:** 1 Department of Chemistry, University of Oxford, Physical and Theoretical Chemistry, South Parks Road, Oxford, OX1 3QZ UK; 2 KU Leuven, Department of Chemistry, Celestijnenlaan 200F, B-3001 Leuven, Belgium; 3 University of Birmingham, Edgbaston, B15 2TT UK

**Keywords:** Chemical physics, Reaction kinetics and dynamics

## Abstract

Isotopic substitution has long been used to understand the detailed mechanisms of chemical reactions; normally the substitution of hydrogen by deuterium leads to a slower reaction. Here, we report our findings on the charge transfer collisions of cold ${{\rm{Xe}}}^{+}$ ions and two isotopologues of ammonia, ${{\rm{NH}}}_{3}$ and ${{\rm{ND}}}_{3}$. Deuterated ammonia is found to react more than three times faster than hydrogenated ammonia. Classical capture models are unable to account for this pronounced inverse kinetic isotope effect. Moreover, detailed ab initio calculations cannot identify any (energetically accessible) crossing points between the reactant and product potential energy surfaces, indicating that electron transfer is likely to be slow. The higher reactivity of ${{\rm{ND}}}_{3}$ is attributed to the greater density of states (and therefore lifetime) of the deuterated reaction complex compared to the hydrogenated system. Our observations could provide valuable insight into possible mechanisms contributing to deuterium fractionation in the interstellar medium.

## Abstract (web summary)

Inverse kinetic isotope effects (KIEs) are rarely observed. Here, the authors report an inverse KIE in the charge transfer reaction of ${{\rm{Xe}}}^{+}$ with ammonia. ${{\rm{ND}}}_{3}$ reacts more than 3 times faster than ${{\rm{NH}}}_{3}$, despite no N-H or N-D bonds being broken, with implications for our understanding of interstellar chemistry.

## Introduction

As long ago as 1932—the year that spectroscopic evidence confirmed the existence of deuterium^1^—Cremer and Polanyi postulated that species containing hydrogen and deuterium would exhibit different reactivities owing to their differences in zero point energy (ZPE)^2^. In the decades that followed this prediction, chemists have exploited the kinetic isotope effect (KIE) to successfully study a plethora of reaction mechanisms. Two key factors that give rise to the deuterium KIE are ZPE considerations and differences in the probability of tunnelling through an energetic barrier. As a result of these factors, one expects a bond involving a D atom to be less reactive than the analogous bond involving an H atom; deuterium-containing species are typically more stable and exhibit lower reaction rate constants than comparable hydrogen-containing isotopologues.

Polanyi himself identified a hypothetical scenario where an exception to the expected deuterium KIE might arise: an inverse isotope effect could occur in processes involving the reaction of atomic H or D^3^. This is because the ZPE of the reactants will be the same, but the deuterium-containing reaction complex will have a lower ZPE. As such, the reaction involving D atoms will require less activation energy to surmount the barrier and form products than the comparable process with H atoms. It should be noted that tunnelling will always be faster for H atoms, which serves to counteract the activation energy decrease for the reaction of atomic D.

True inverse primary KIEs in single-step processes are seldom observed experimentally. The reductive elimination of transition metal hydride complexes has seen a number of inverse primary KIEs identified—although several of these inverse KIEs (reported for overall reductive elimination processes) have subsequently been attributed to an inverse equilibrium isotope effect in the rate-determining step, rather than a true single-step inverse primary KIE^4^. Inverse secondary KIEs, where the isotopic substitution is made in a bond that does not break during the reaction, have been seen in reactions that involve changes to the hybridization of one or more carbon atoms in organic molecules^5,6^. However, inverse secondary KIEs tend to be much less pronounced than inverse primary KIEs, with the ratio of the reaction rate constants for the hydrogenated and deuterated processes typically falling within the range ${k}_{{\rm{H}}}/{k}_{{\rm{D}}}=0.8-0.9$^6^.

In addition to enabling one to probe reaction mechanisms, examining the reactions of small deuterated molecules is directly relevant to the chemistry of the interstellar medium (ISM). There is a higher abundance of deuterium in molecules detected in the ISM than would be expected from the cosmic D/H ratio ($\approx 1.5\times 1{0}^{-5}$), a phenomenon known as deuterium fractionation^7^. Modelling deuterium fractionation requires the consideration of a series of increasingly complex reaction systems, and must also account for the interplay between gas-phase and grain-surface chemistry^8,9^. The prevailing low temperatures and low particle densities of the ISM magnify the significance of any small endothermicities, differences in ZPE, and the mass dependence of centrifugal barriers. There are very few experimental studies of astrochemically important reaction systems under relevant conditions^10^. Measurements of reaction rate constants in such systems are essential for the development of a comprehensive model of the competing processes taking place in the ISM^11^.

In this work, we present results on a charge exchange reaction involving state-selected sympathetically cooled ${{\rm{Xe}}}^{+}$ ions and thermal ${{\rm{NH}}}_{3}$ or ${{\rm{ND}}}_{3}$ neutral molecules. We observe a significant inverse KIE, with ${k}_{{\rm{H}}}/{k}_{{\rm{D}}}=0.3$; fully deuterated ammonia (${{\rm{ND}}}_{3}$) charge exchanges with ${{\rm{Xe}}}^{+}$ more than three times faster than the fully hydrogenated analogue (${{\rm{NH}}}_{3}$). As no N–H or N–D bonds are broken in the course of the reaction, the presence of a ${k}_{{\rm{H}}}/{k}_{{\rm{D}}}$ ratio of 0.3 is remarkable. We are not aware of any comparable systems where such a pronounced inverse KIE has been reported. The higher reactivity of deuterated ammonia cannot be explained by simple capture theory models. Both ${{\rm{ND}}}_{3}$ and ${{\rm{NH}}}_{3}$ have been observed in the ISM^12^. Reactions between ions and polar neutral molecules are known to be astrochemically important, as such processes generally proceed with no activation energy barrier and exhibit rate constants with an inverse temperature dependence. Our measurement of a significant inverse KIE in a simple charge exchange reaction could unveil a mechanism that is important in its own right, as well as being of potential astrochemical interest.

## Results

### Experimental set-up

**Fig. 1:**
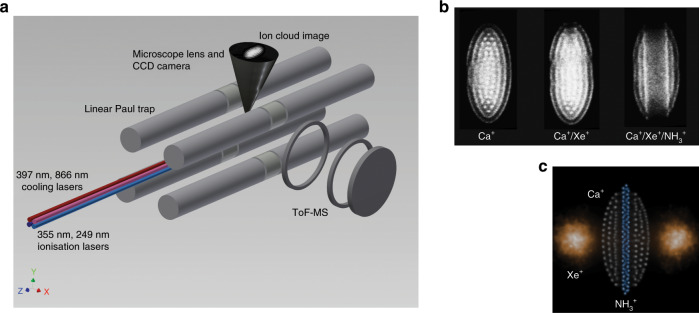
Experimental set-up. **a** Coulomb crystals are formed when trapped ${{\rm{Ca}}}^{+}$ ions are laser cooled. The lattice positions of the constantly fluorescing ${{\rm{Ca}}}^{+}$ ions can be directly observed by a charge-coupled device (CCD) camera, with the presence of other co-trapped species inferred from changes to the ${{\rm{Ca}}}^{+}$ fluorescence pattern. Time-of-flight (ToF) traces are recorded by ejecting the ions from the trap and onto a multichannel plate (MCP) detector. See the “Methods” section for further details. **b** Experimental images of mono-component (${{\rm{Ca}}}^{+}$-only, left), bi-component (${{\rm{Ca}}}^{+}$ and ${{\rm{Xe}}}^{+}$, centre) and tri-component (${{\rm{Ca}}}^{+}$, ${{\rm{Xe}}}^{+}$, and ${{\rm{NH}}}_{3}^{+}$, right) Coulomb crystals, showing the progress of the charge exchange reaction. **c** False-colour image of a simulated tri-component Coulomb crystal, illustrating the locations of the non-fluorescing ${{\rm{NH}}}_{3}^{+}$ and ${{\rm{Xe}}}^{+}$ ions in addition to the ${{\rm{Ca}}}^{+}$ ions. See Supplementary Note 3 for details on the simulations.

Probing the reaction dynamics of elementary chemical reactions has been a long-standing challenge for experimentalists. Coulomb crystals are increasingly being utilised to study the dynamics and kinetics of ion-neutral reactions under controlled conditions. In this work, ${{\rm{Xe}}}^{+}$ ions are created by laser ionisation, and are sympathetically cooled into the framework of a Coulomb crystal that consists of a few hundred laser-cooled ${{\rm{Ca}}}^{+}$ ions held within a linear Paul ion trap. The ${{\rm{Xe}}}^{+}$ ions are allowed to react (charge transfer) with a thermal sample of ammonia gas, and reactions are monitored by (indirectly) observing the appearance of ammonia ions. The many advantages of using Coulomb crystals for the study of reaction processes, and the details of the ion trap system used in this work, have been described elsewhere^13–23^. Combining laser-induced fluorescence images with time-of-flight (ToF) mass spectrometry (MS) allows for the quantitative calculation of reaction rate constants for the systems of interest, namely ${{\rm{Xe}}}^{+}$ (${}^{2}{{\rm{P}}}_{3/2}$) + ${{\rm{NH}}}_{3}$(${}^{1}{{\rm{A}}}_{1}$) and ${{\rm{Xe}}}^{+}$(${}^{2}{{\rm{P}}}_{3/2}$) + ${{\rm{ND}}}_{3}$(${}^{1}{{\rm{A}}}_{1}$) (see Fig. 1).

### Reaction rate constant measurements

The rate constant measured in this work for the charge transfer reaction between ${{\rm{Xe}}}^{+}$ and ${{\rm{NH}}}_{3}$ is ${k}_{{\rm{H}}}=0.36(2)\times 1{0}^{-9}$ ${{\rm{cm}}}^{3}$ ${{\rm{s}}}^{-1}$; for the reaction between ${{\rm{Xe}}}^{+}$ and ${{\rm{ND}}}_{3}$, the rate constant is ${k}_{{\rm{D}}}=1.2(2)\times 1{0}^{-9}$ cm^3^ s^−1^. The reactivity of ${{\rm{NH}}}_{3}$ and ${{\rm{ND}}}_{3}$ is thus significantly different, resulting in an inverse KIE of ${k}_{{\rm{H}}}/{k}_{{\rm{D}}}=0.3$. As no bonds are broken during the charge exchange reaction, only a minor normal KIE (if any discernible difference at all) is expected when comparing the reactions.

**Fig. 2:**
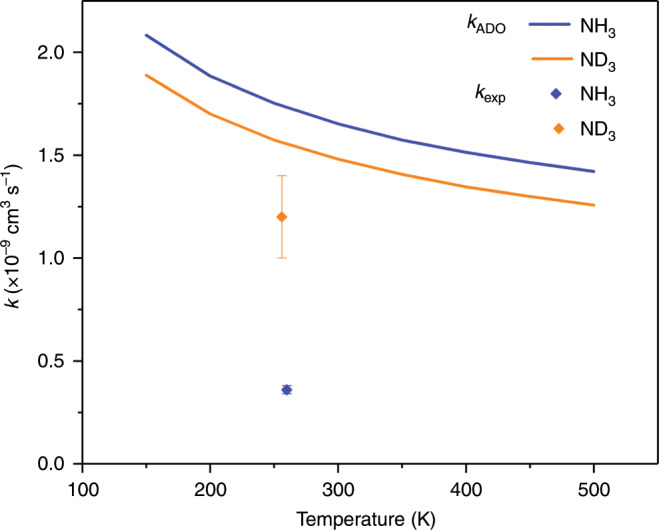
Charge exchange rate constants. Comparison between experimentally measured reaction rate constants and those predicted by the ADO model for the charge exchange reaction between ${{\rm{Xe}}}^{+}$ and ${{\rm{NH}}}_{3}$ (blue) or ${{\rm{ND}}}_{3}$ (orange). Error bars indicate the uncertainty in the experimental rate constants. See Supplementary Note 4, Supplementary Fig. 1 and Supplementary Table 2 for further details.

Average dipole orientation (ADO) theory^24,25^ is a capture model developed for calculating the reactions of ions with polar neutral molecules. The ADO model extends Langevin’s theory by taking into account the properties of the neutral polar reactant: a term that accounts for the temperature of the system and the permanent dipole moment of the polar molecule is added to the Langevin equation. (See Supplementary Note 5 and Supplementary Table 3 for full details on the ADO calculations.) As the population of rotational states in ammonia is thermal in our experiments (290 K), the ADO model is considered a suitable classical approximation for this study. Calculations performed using ADO theory predict that the reaction rate constants for the two isotopologues should be very similar, with a small normal KIE of ${k}_{{\rm{H}}}/{k}_{{\rm{D}}}=1.1$ anticipated (see Table 1). This small difference in the expected rate constants arises from differences in the polarisability and dipole moment of the isotopologues^26–28^, in addition to their different masses. Not only is the magnitude of the predicted ADO rate constants larger than in our measurements (see Fig. 2 and Table 1), we record a significant inverse KIE.

**Table 1:** ADO and experimental rate constants for the two ammonia isotopologues of interest.

Isotopologue	${k}_{{\rm{ADO}}}$ (${{\rm{cm}}}^{3}$ ${{\rm{s}}}^{-1}$)	${k}_{\exp }$ (${{\rm{cm}}}^{3}$ ${{\rm{s}}}^{-1}$)
${{\rm{NH}}}_{3}$	$1.8\times 1{0}^{-9}$	$0.36(2)\times 1{0}^{-9}$
${{\rm{ND}}}_{3}$	$1.6\times 1{0}^{-9}$	$1.2(2)\times 1{0}^{-9}$

**Fig. 3:**
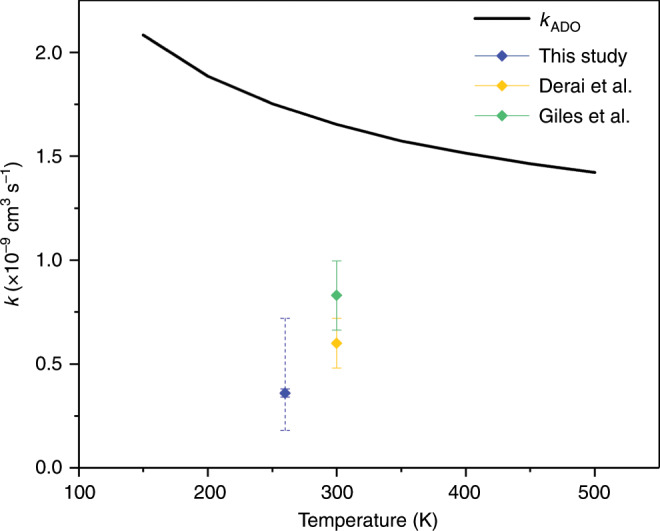
Comparison between $${{\rm{Xe}}}^{+}$$ + $${{\rm{NH}}}_{3}$$ charge exchange rate constants. Reaction rate constants obtained from the ADO model (black line) and from several experimental measurements are plotted for the $${{\rm{Xe}}}^{+}$$$$+$$ $${{\rm{NH}}}_{3}$$ reaction. The result from Derai et al.^29^ is provided in yellow, Giles et al.^30^ is in green (both at 300 K) and our result (at 260 K) is plotted in blue. All of the experimental reaction rate constants are lower than the ADO model prediction. Note that a second (dashed) error bar is included in the rate constant established in this work. The additional error bar is to allow for the factor of two uncertainty in the partial pressure readings (as these were taken using a pressure gauge) for comparison with the previous studies.

The temperature assigned to each reaction is taken as a weighted average of the temperature of the ammonia neutrals (290 K) and the average translational temperature of the sympathetically cooled $${{\rm{Xe}}}^{+}$$ ions ($$\sim$$30 K, established from molecular dynamics simulations and arising due to the secular motion and micromotion of the ions). This approach yields a temperature of 260 K for the $${{\rm{Xe}}}^{+}$$$$+$$ $${{\rm{NH}}}_{3}$$ reaction and 256 K for the $${{\rm{Xe}}}^{+}$$$$\,+\,$$ $${{\rm{ND}}}_{3}$$ process. Previous studies on the $${{\rm{Xe}}}^{+}$$$$+$$ $${{\rm{NH}}}_{3}$$ system at 300 K, conducted using an ion cyclotron resonance (ICR) cell^29^ and a selected-ion flow tube (SIFT)^30^, reported reaction rate constants that are comparable to our experimental findings (plotted in Fig. 3). The findings of these previous experimental studies are also somewhat lower than the ADO model prediction (see Fig. 3). We know of no previous experimental studies on the $${{\rm{Xe}}}^{+}$$$$+$$ $${{\rm{ND}}}_{3}$$ system.

Interestingly, studies conducted on the $${{\rm{Xe}}}^{+}$$$$+$$ $${{\rm{NH}}}_{3}$$ reaction at hyperthermal collision energies exhibit excellent agreement with ADO theory over a higher temperature range, from ~0.06 eV (700 K) to 34 eV^31^, in contrast to the measurements taken at $$T\le 300$$ K. Such behaviour indicates that a more complex model of the reaction system is required to understand the mechanism of charge exchange under atmospherically and astrochemically relevant conditions.

### Other reaction pathways

The presence of additional reaction pathways that might be competing with the charge exchange process, such as hydrogen abstraction, $${{\rm{Xe}}}^{+}$$$$+$$ $${{\rm{NH}}}_{3}$$$$\to$$$${{\rm{XeH}}}^{+}+{{\rm{NH}}}_{2}$$, has been considered as a possible explanation for the observed inverse KIE. However, only the charge transfer pathway is exothermic: $${{\rm{Xe}}}^{+}$$$$+$$ $${{\rm{NH}}}_{3}$$$$\to$$Xe + $${{\rm{NH}}}_{3}^{+}$$, $$\Delta H\ =\ -2.1$$ eV; all other reaction pathways (including the hydrogen abstraction reaction specified above) have endoergicities $$> 1$$ eV^31^. Furthermore, ToF-MS recorded after completion of the reaction indicate that only $${{\rm{Ca}}}^{+}$$ and ammonia ions are present in the crystal, excluding the formation of $${{\rm{XeH}}}^{+}$$ (or $${{\rm{XeD}}}^{+}$$) from hydrogen (deuterium) abstraction. As such, we conclude that no competing reactions are occurring under our experimental conditions (see Supplementary Note 1 for a more in-depth discussion).

## Discussion

Ion–molecule reactions in the gas-phase are often described as barrierless. The lack of a reaction energy barrier arises from the attractive long-range ion–dipole or ion-induced–dipole forces dominating the potential energy profile in the entrance channel, overcoming the short-range energy hill due to the initial breaking of bonds. However, a centrifugal barrier is created for collisions in any configuration other than head on, associated with the orbital angular momentum of the collision partners around one another. Capture theories assume that for collisions that have sufficient energy to surpass the centrifugal barrier, the partners come together to form an ion–molecule complex and the reaction (in the present case, an electron transfer reaction) occurs subsequently with 100% probability—hence, the rate is determined by the rate of formation of the complex. This implies that the reaction occurs in a much shorter time frame than the lifetime of the complex with respect to dissociating back to reactants. Observed rates that are lower than those predicted by capture theory, as reported in the current work, suggest that the reaction does not proceed with 100% probability following complex formation.

For a simple ion–atom collision (under low-pressure conditions), the complex formed has a very short lifetime—of the order of one vibrational period—because there is nothing to stop the collision partners rebounding off the inner repulsive wall and re-dissociating. For ion–molecule collisions, the lifetime of the complex is longer because the kinetic energy initially directed along the collision co-ordinate (the ion–molecule distance) can be dispersed into other low-energy vibrational modes of the complex—a process known as intramolecular vibrational redistribution (IVR). Dissociation of the complex does not occur until sufficient energy has found its way back into the bond-breaking co-ordinate. For the [${{\rm{NH}}}_{3}$–Xe]${}^{+}$ complex, the energy could be diverted into the (hindered) internal rotation of the ${{\rm{NH}}}_{3}$ moiety within the complex, or the rocking modes of the complex (with the ${{\rm{NH}}}_{3}$ tilting relative to the Xe–N axis), or into the vibrational modes of the ${{\rm{NH}}}_{3}$ moiety itself (for example, bond stretching or the scissors motion of two of the N–H bonds), or the umbrella bend (although the full inversion in this mode will be hindered by the presence of the ion). All of these modes will have a lower frequency for deuterated ammonia, owing to the motion of the D/H atoms relative to the centre of mass.

It is well established that the rate of IVR in polyatomic molecules and complexes depends on the density of vibrational states within the complex (see, for example, refs. ^32–35^). It is also known that deuteration can lead to faster IVR, because the greater mass of deuterium reduces vibrational frequencies, increasing the density of vibrational states at a given energy^36–39^. Not only is the rate at which the energy is dispersed by IVR likely to be greater for the deuterated complex, but also (because the energy is dispersed into a greater number of vibrational states), the statistical rate at which the energy finds its way back into the dissociation co-ordinate is lower. Hence, the lifetime of the deuterated complex is expected to be longer than that of the hydrogenated complex. We should stress that this is only a speculative explanation for our experimental observations; the extent to which one can use statistical theories of reactivity to describe the properties of the charge exchange between ${{\rm{Xe}}}^{+}$ and ${{\rm{NH}}}_{3}$ or ${{\rm{ND}}}_{3}$ remains to be seen.

This picture of the long-lived reaction complex, with energy sequestered in the numerous internal degrees of freedom and taking time to localise into the relevant mode(s) for product formation, is reminiscent of the roaming reaction mechanism. First observed in formaldehyde photodissociation^40^, roaming reaction pathways have subsequently been identified in a number of other systems^41–46^. Very recently, Willitsch and co-workers identified a roaming-like reaction pathway in the charge exchange of $${{\rm{N}}}_{2}^{+}$$ with Rb^47^. The roaming-like mechanism observed in $${{\rm{N}}}_{2}^{+}$$$$+$$ Rb involved the Rb atom orbiting around the central $${{\rm{N}}}_{2}$$ core of the reaction complex, undergoing several intramolecular collisions until the orientation and distribution of energy in the two moieties was such that the reaction could proceed to products^47^. A defining characteristic of roaming reaction pathways is the presence of long-range attractive forces in the reaction intermediate. This behaviour is also seen in the intermediates formed in ion–molecule reactions, where ion-dipole forces hold the reaction complex together^48^. As a result of these common long-range attractive features, roaming reaction pathways are expected to play a role in ion–molecule reactions^49^. A signature of roaming reaction processes can be found in the way that energy is distributed into the product degrees of freedom. In order to unambiguously ascertain what role—if any—roaming reaction mechanisms play in the charge exchange of $${{\rm{Xe}}}^{+}$$ with $${{\rm{NH}}}_{3}$$ or $${{\rm{ND}}}_{3}$$, further experimental work or more detailed theoretical modelling must be undertaken (for example, to probe the product state distribution of the ammonia ions).

**Fig. 4:**
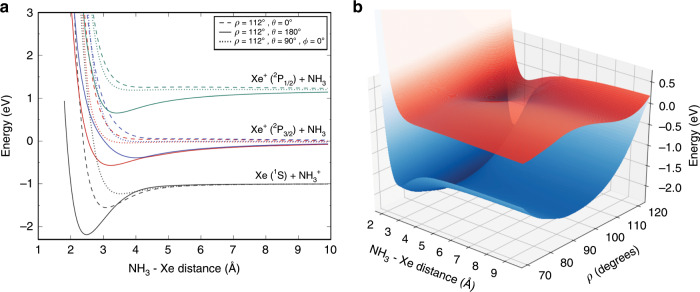
Potential energy surfaces. **a** A series of one-dimensional cuts are plotted as a function of the distance $R$ between Xe and the center of mass of ${{\rm{NH}}}_{3}$, for the four PESs of the [Xe–${{\rm{NH}}}_{3}$]^+^ complex that are relevant to the charge transfer process. Each of the PESs is plotted in a different colour. Three different angles of approach are shown for each surface, with the umbrella angle fixed to the equilibrium value of ${{\rm{NH}}}_{3}$, $\rho =11{2}^{\circ }$. The preferred orientation ($\theta =18{0}^{\circ }$) corresponds to ${{\rm{Xe}}}^{+}$ approaching the N atom end of ammonia along its symmetry axis. (Please see the “Methods” section for further details.) **b** PESs of the two lowest molecular states, shown for the most favourable orientation ($\theta =18{0}^{\circ }$) and plotted as a function of $R$ and $\rho$. The upper surface corresponds to the ${{\rm{Xe}}}^{+}$ + ${{\rm{NH}}}_{3}$ reactants (the entrance channel), with the lower surface representing the Xe $+$ NH${}_{3}^{+}$ products (the exit channel). In the upper PES, the double well of ${{\rm{NH}}}_{3}$ is seen to be strongly perturbed by the presence of the ${{\rm{Xe}}}^{+}$ ion. At large umbrella angles, the two surfaces become almost degenerate.

The most favourable orientation for a reactive collision is with the negative end of the dipole (N atom and lone pair) pointing towards the ion. This corresponds to an angle of $\theta =18{0}^{\circ }$, as illustrated in Fig. 4, which shows one-dimensional cuts through the potential energy surfaces (PESs) for the ${{\rm{Xe}}}^{+}$ + ${{\rm{NH}}}_{3}$ system (see “Methods” section for full details on the calculations). Electron transfer involves the non-adiabatic hopping of the system from an upper surface onto the lower surface, with conservation of energy. We expect that electron transfer will be intrinsically slow for this reaction, as there is a large change in equilibrium geometry that must take place between the neutral ${{\rm{NH}}}_{3}$ molecule and the ${{\rm{NH}}}_{3}^{+}$ ion; the former is pyramidal while the latter is planar at equilibrium.

The charge transfer process is highly exoergic (by 2.1 eV). It is expected that electron transfer will be fast if there is a crossing between the PESs representing the initial and final states of the transfer, in an energetically accessible region. The calculations shown in Fig. 4 suggest that there is no such crossing, and we are unable to identify such a crossing in calculations for other geometries of the ${{\rm{NH}}}_{3}$ moiety or orientations of the reaction partners. Although crossings may appear for excited vibrational states, we expect the electron transfer to be relatively slow because of the poor Franck Condon overlap of the wavefunctions in the dissociation co-ordinate for the initial and final states. While charge transfer is slower than anticipated by a simple capture theory formalism, on an absolute scale the charge exchange reactions proceed surprisingly quickly—especially given the absence of a curve crossing. As the ${{\rm{NH}}}_{3}$ umbrella angle increases, the PESs move closer together in energy, and therefore the Franck Condon factors may become less unfavourable. (Even in the ground vibrational state of the umbrella mode, the ${{\rm{NH}}}_{3}$ wavefunction is non-negligible for umbrella angles >120°, where the PESs become closer in energy.) It is likely, therefore, that IVR into the umbrella bending mode would enhance the probability of electron transfer by increasing the probability that the system reaches this extreme part of the potential energy surface. However, the rate of charge transfer is still expected to be low; the complex stabilisation achieved by IVR is not expected to be substantial, as this is only a five-atom complex with relatively high-frequency vibrational modes. Hence, for both ${{\rm{NH}}}_{3}$ and ${{\rm{ND}}}_{3}$, the charge transfer probability on complex formation is likely to be below 100%.

In summary, the lower-than-anticipated rate constants for both reactions (based on the capture theory predictions) is likely to be a consequence of the low intrinsic rate of electron transfer within the reaction complex. The longer lifetime of the [${{\rm{ND}}}_{3}$–Xe]^+^ complex compared to the [${{\rm{NH}}}_{3}$–Xe]^+^ complex would give a greater probability of the (slow) reaction occurring within the lifetime of the deuterated complex, i.e. before the complex dissociates back to reactants, and thus could account for the observed inverse KIE. Verification of this explanation requires further theoretical investigation, and will be a subject of future work.

## Conclusions

A substantial inverse KIE, ${k}_{{\rm{H}}}/{k}_{{\rm{D}}}=0.3$, is observed in the charge exchange of ${{\rm{Xe}}}^{+}$ with ${{\rm{NH}}}_{3}$ and ${{\rm{ND}}}_{3}$. A classical capture theory treatment of the reaction systems is unable to account for the experimental rate constants. With the aid of ab initio potentials, we predict electron transfer to be slow; the absence of curve crossings in energetically accessible regions implies that the non-adiabatic transition probability is low. The inverse KIE is ascribed to the longer predicted lifetime of the [${{\rm{ND}}}_{3}$–Xe]^+^ reaction complex.

Our proposed explanation has potential implications for deuterium fractionation in the ISM, even though the current reaction under study is not itself one of interstellar importance. The inverse KIE observed here could also feature in other processes involving an ion and a small hydrogenated polyatomic molecule (such as $${{\rm{H}}}_{2}{\rm{O}}$$, $${{\rm{CH}}}_{3}$$, $${{\rm{NH}}}_{3}$$, $${{\rm{C}}}_{2}{{\rm{H}}}_{2}$$). This would be especially applicable to slow electron transfer processes, but could also apply to reactions in which there is a submerged reaction barrier (as observed in previous work on $${{\rm{Ca}}}^{+}$$$$+$$ $${{\rm{CH}}}_{3}$$F)^15^, or in other cases where the reaction in the complex is not intrinsically fast.

## Methods

### Experimental methods

Ion-neutral reactions are studied within the confines of a Coulomb crystal, held in a linear Paul ion trap. The experiments are conducted under ultra-high vacuum conditions, with the set-up detailed in previous work^14,15^. Four cylindrical Paul trap electrodes, each separated into three segments, are held in a quadrupole arrangement (see Fig. 1). A cosine radio frequency (RF) voltage of the form $\pm \!\frac{1}{2}{V}_{{\rm{RF}}}$cos$({\Omega }_{{\rm{RF}}}t)$ (where ${V}_{{\rm{RF}}}$ is the peak-to-peak voltage amplitude and ${\Omega }_{{\rm{RF}}}$ is the RF drive frequency) is applied to the electrodes and produces a time-dependent trapping potential in the radial plane. An additional static potential (${U}_{{\rm{DC}}}$) is applied across the eight end-cap segments, for axial confinement of the ions. The trapping parameters employed in this study are ${V}_{{\rm{RF}}}=200$ V, ${U}_{{\rm{DC}}}=2.7$ V and ${\Omega }_{{\rm{RF}}}=2\pi \times 3.822$ MHz. The trap has dimensions ${r}_{0}=3.5$ mm and ${z}_{0}=2.75$ mm (with ${r}_{0}$ half the diagonal electrode surface separation and ${z}_{0}$ half the end-cap separation). ${{\rm{Ca}}}^{+}$ ions are formed in the trap by non-resonant ionisation of an effusive, skimmed calcium atom beam directed toward the trap centre, using the tripled output of a Nd:YAG laser at 355 nm. ${{\rm{Ca}}}^{+}$ ions are cooled by two diode lasers, which address the main cooling transition ($4{s}\,^{2}{{\rm{S}}}_{1/2}\to$ $4{p}\,^{2}{{\rm{P}}}_{1/2}$, 397 nm) and a re-pumping transition ($3{d}\,^{2}{{\rm{D}}}_{3/2}\to$ $4{p}\,^{2}{{\rm{P}}}_{1/2}$, 866 nm). The fluorescence emitted by laser-cooled ${{\rm{Ca}}}^{+}$ ions is focused by a ×10 objective lens into a charge-coupled device (CCD) camera, yielding a two-dimensional image of the central slice of the Coulomb crystal in real time.

Xenon gas is admitted into the trap chamber using a high-precision leak valve and is ionised by the doubled output of a Nd:YAG-pumped dye laser (at 249.6 nm) directed into the trap centre, via a (2 + 1) resonance enhanced multi-photon ionisation (REMPI) scheme. The resulting ${{\rm{Xe}}}^{+}$ ions are sympathetically cooled by the laser-cooled ${{\rm{Ca}}}^{+}$ ions, forming a bi-component Coulomb crystal. A time delay of 60 s is introduced at this stage, to ensure that all ${{\rm{Xe}}}^{+}$ ions have been thermalised (through elastic collisions with the ${{\rm{Ca}}}^{+}$ ions) and are in the desired ${}^{2}{{\rm{P}}}_{3/2}$ ground state. Approximately 22% of ${{\rm{Xe}}}^{+}$ ions are formed in a higher-energy ${}^{2}{{\rm{P}}}_{1/2}$ spin state with this REMPI scheme^50^. Although the ${}^{2}{{\rm{P}}}_{1/2}\to$ ${}^{2}{{\rm{P}}}_{3/2}$ transition is electric dipole forbidden, the magnetic dipole transition is allowed with an Einstein coefficient of ${A}_{m}$ = 21 ${{\rm{s}}}^{-1}$, resulting in an upper state lifetime of ~48 ms^51^. By introducing a delay of 60 s, we ensure that all ${{\rm{Xe}}}^{+}$ ions are in the energetically lower-lying ${}^{2}{{\rm{P}}}_{3/2}$ spin state prior to undergoing reaction with ammonia.

Room temperature (290 K) ammonia molecules (${{\rm{NH}}}_{3}$ or ${{\rm{ND}}}_{3}$) are introduced to the reaction chamber through a second high-precision leak valve, initiating the charge exchange reaction ${{\rm{Xe}}}^{+}+{{\rm{NH}}}_{3}\to$ Xe + ${{\rm{NH}}}_{3}^{+}$ (or the deuterated analogue). Reactions take place at ammonia partial pressures of $3\times 1{0}^{-9}$ mbar. The lighter ammonia product ions produced by the charge exchange reaction displace the fluorescing ${{\rm{Ca}}}^{+}$ ions from the centre of the crystal and form a dark core. The growth of the dark core qualitatively indicates the progress of the reaction (see Fig. 1, Supplementary Note 2 and Supplementary Table 1). Rate constants for each reaction are calculated by analysing crystal composition as a function of time, and are complemented by ToF-MS recorded at the end of each reaction. Charge exchange between xenon ions and ammonia molecules is a one-to-one process, thus d[${{\rm{NH}}}_{3}^{+}$]/d$t$ = −d[${{\rm{Xe}}}^{+}$]/d$t$. Assuming a constant density of ammonia reactant molecules, this pseudo-first-order process can be expressed as ${[{{\rm{Xe}}}^{+}]}_{t}$ = ${[{{\rm{Xe}}}^{+}]}_{0}{{\mathrm{{e}}}}^{-{k}_{1}t}$, or ${[{{\rm{NH}}}_{3}^{+}]}_{t}$ = ${[{{\rm{Xe}}}^{+}]}_{0}(1-{{\mathrm{{e}}}}^{-{k}_{1}t})$, with ${[{{\rm{Xe}}}^{+}]}_{0}$ the initial number density of ${{\rm{Xe}}}^{+}$ ions and ${k}_{1}$ the pseudo-first-order rate constant. A comprehensive discussion of the calculation of the rate constants can be found in Supplementary Note 4.

### Theoretical methods

PESs have been constructed for the Xe–${{\rm{NH}}}_{3}^{+}$ system by means of the multi-configurational self-consistent field method (MCSCF) followed by multi-reference configuration interaction (MRCI) calculations, including the Davidson correction, as implemented in the MOLPRO quantum chemistry package^52–54^. As the N–H bond length is similar for ${{\rm{NH}}}_{3}$ and ${{\rm{NH}}}_{3}^{+}$, this distance is kept fixed at 1.92${a}_{0}$. The PESs therefore depend on four coordinates: $R$ is the length of the vector ${\bf{R}}$ describing the position of the Xe^(+)^ atom with respect to the center of mass of ${{\rm{NH}}}_{3}^{(+)}$, $\theta$ (with ${0}^{\circ }\le \theta ^{\prime} \le 18{0}^{\circ }$) is the angle between the vector ${\bf{R}}$ and the ${{\rm{C}}}_{3}$ axis, and $\varphi$ (with ${0}^{\circ }\le \varphi ^{\prime} \le 36{0}^{\circ }$) is the angle of rotation of this vector around the ${{\rm{C}}}_{3}$ axis. ${{\rm{Xe}}}^{+}$ approaches on the N atom end of the ammonia molecule for $\theta =18{0}^{\circ }$. Finally, the angle $\rho$ describes the umbrella motion of ammonia. For ${{\rm{NH}}}_{3}$, the equilibrium value is $\rho =112.{1}^{\circ }$; ${{\rm{NH}}}_{3}^{+}$ is planar, with $\rho =9{0}^{\circ }$.

The charge transfer dissociation channels Xe + ${{\rm{NH}}}_{3}^{+}$ and ${{\rm{Xe}}}^{+}$ + ${{\rm{NH}}}_{3}$ correspond to the ground and first excited states of the complex, respectively. There are no other molecular states with energy between the two charge transfer channels. The Xe(${}^{2}{\rm{S}}$) + ${{\rm{NH}}}_{3}^{+}$(${}^{2}{{\rm{A}}}_{2}^{^{\prime\prime} }$) channel leads to a ${}^{2}{{\rm{A}}}_{1}$ state in the ${{\rm{C}}}_{3v}$ point group, while the ${{\rm{Xe}}}^{+}$(${}^{2}{\rm{P}}$) + ${{\rm{NH}}}_{3}$(${}^{1}{{\rm{A}}}_{1}$) channel gives rise to a ${}^{2}{{\rm{A}}}_{1}$ state and a doubly degenerate ^2^E state. The ab initio calculations are performed in the subgroups ${{\rm{C}}}_{s}$ or ${{\rm{C}}}_{1}$. In ${{\rm{C}}}_{s}$, the relevant molecular states for the charge-transfer process become three ${}^{2}{\rm{A}}^{\prime}$ states and one ${}^{2}{\rm{A}}^{\prime\prime}$ state.

The aug-cc-pVTZ basis set is used for all atoms. The 28 inner electrons of the Xe atom are described by an effective core potential (ECP28MDF). The $4s$, $4p$ and $4d$ orbitals of Xe as well as the $1{s}^{2}$ orbital of N are kept frozen. The active space therefore consists of the remaining orbitals: $5{s}^{2}5{p}^{6}$ for Xe, $2{s}^{2}{p}^{3}$ for N, and $1s$ for each H atom. In the ${{\rm{C}}}_{s}$ point group, the total number of orbitals used to describe the 35 electrons is ($7a^{\prime} +3a^{\prime\prime}$) core orbitals and ($8a^{\prime} +3a^{\prime\prime}$) additional orbitals in the active space.

The spin–orbit (SO) coupling in ${{\rm{Xe}}}^{+}$ is large (10,537 cm^−1^) and is included in the present calculations by diagonalising the Breit–Pauli operator on the basis of MRCI wavefunctions. Its effect is to split the degenerate excited states of the complex into two states with $\Omega =3/2$ that are degenerate at dissociation and a $\Omega =1/2$ state, 10,537 cm^−1^ higher at dissociation. The ground state of the molecular complex is almost unaffected by the SO interaction.

In order to scan the potential energy surface landscape, the energies of the four molecular states are computed for 15 values of $R$ between 2 and 6 Å, 6 values of $\rho$ between $9{0}^{\circ }$ and $12{5}^{\circ }$, 9 values of $\theta$ and three values of $\varphi$. For the particular approach paths corresponding to $\theta ={0}^{\circ }$ (Xe along the ${{\rm{C}}}_{3}$ axis on the side of the H atoms), $\theta =18{0}^{\circ }$ (approach on the N atom end), and $\theta =9{0}^{\circ }$ (with $\varphi ={0}^{\circ }$ or $\varphi =6{0}^{\circ }$), a more extensive grid is explored—with 28 values of $R$ between 1.8 and 10 Å, and 10 values of $\rho$ between 90° and 123°. The most favourable orientation for reaction occurs when the ${{\rm{Xe}}}^{+}$ ion approaches the ammonia molecule from the N atom end, as shown in Fig. 4.

## Supplementary information

Supplementary Information

## Data Availability

Supporting data can be obtained from the Oxford Research Archive, 10.5287/bodleian:NoRj6KRde.
