# Supplementary material for: Strong inverse kinetic isotope effect observed in ammonia charge exchange reactions
Source: Nat Commun. 2020 Jan 10;11:173. doi: 10.1038/s41467-019-13976-8 (PMC6954264; doi:10.1038/s41467-019-13976-8)
Supplement: Supplementary file 1 — Supplementary Information [file 41467_2019_13976_MOESM1_ESM.pdf]

Supplementary Information

Strong inverse kinetic isotope effect observed in ammonia charge exchange reactions

Petralia et al.

Supplementary Note 1. Treatment of background reactions

The Ca^+ laser-induced fluoresence pattern can provide information on whether there are species other than the laser-cooled Ca^+ ions within the trap. While one can deduce whether these co-trapped species have a mass-to-charge ratio that is higher or lower than Ca^+, it is not possible to unambiguously identify non-laser-cooled species from imaging alone. It is therefore essential that any contaminants—and any competing reaction processes—are eliminated (or at least minimised and quantified) before the reaction of interest can be quantitatively studied. Any gaseous contaminants present in the chamber can be identified using a residual gas analyser. To reduce the probability of trapped ions undergoing reactive collisions with background contaminant species, a heat exchange device is operated. Liquid-nitrogen-cooled N_2 gas passes through a series of turns of tubing, resulting in the freezing out of any gaseous contaminants present in the chamber. Prior to the recording of experimental measurements, N_2 gas is constantly flowed through the heat exchanger for at least two hours, reducing the amount of contaminants present to baseline levels. This process is also accompanied by a decrease in the pressure of the reaction chamber (to below 1.5×10^{-9} mbar).

Any ions produced by background reactions can be monitored via the presence of contaminant peaks in the time-of-flight mass spectra (ToF-MS). Background reaction studies of mono-component Ca^+ and bi-component Ca^+/Xe^+ crystals show that the Xe^+ ions react with contaminant species, such as O_2 or pump-oil, at a faster rate than the Ca^+ ions. On the timescale of the charge exchange reactions of interest, however, background reactions are either absent or negligible, as determined from the ToF-MS data. In some cases, a small number of Xe^+ ions are found to react with residual ammonia neutrals in the chamber. This is demonstrated by the formation of a dark core prior to the admission of ammonia reactants into the chamber. In cases where this occurs, the reaction between Xe^+ ions and residual background ammonia molecules is quantified by molecular dynamics (MD) simulations and accounted for in the resulting analysis (see Supplementary Note 4).

Supplementary Note 2. Evaluation of the charge exchange reaction as a one-to-one process

The charge exchange reaction between Xe^+ ions and NH_3 or ND_3 molecules is a one-to-one process. Previous studies on the $Xe^+ + NH_3$ system have identified that only the charge transfer pathway is exothermic ($\Delta H = -2.1$ eV) for the $Xe^+(^2P_{3/2})$ and $NH_3(^2A_1)$ species of interest in this work.[1] Other reaction pathways, such as hydrogen abstraction, have endoergicities >1 eV. Additionally, no unassigned peaks are observed in the ToF-MS after completion of the reaction, further excluding the formation of (for example) XeH^+ products. To the best of our knowledge, there are no comparable studies on the deuterated ammonia system. With the absence of any XeD^+ peaks in the ToF traces, and the high likelihood that the formation of XeD^+ ions is similarly endothermic, we are confident that there are no other competing reaction pathways for the analogous charge exchange process with ND_3 under our experimental conditions. As such, given that the rate of any reactions with background gases is negligible and there are no competing reaction pathways open, we assume that every Xe^+ ion that reacts produces one ammonia ion.

The trapping efficiency of the ammonia ions after their formation must also be considered. The trap depth for a given ion is calculated from the equation

$$\phi(r_0) = \frac{Q^2 V_{\rm RF}^2}{4m\Omega_{\rm RF}^2 r_0^2} - \frac{\eta Q U_{\rm DC} r_0^2}{2z_0^2}, \tag{1}$$

where Q is the charge of the ion, $V_{\rm RF}$ the peak-to-peak amplitude of the radio-frequency oscillating field, m the mass of the ion, $\Omega_{\rm RF}$ the frequency of the oscillating field and $U_{\rm DC}$ the static end-cap voltage. Parameters r_0, η and z_0 are related to the trap geometry. The first and second terms of Supplementary Equation (1) represent the radial ($x - y$ plane) and axial (z-axis) depth of the trap, respectively. Supplementary Table I summarises the trap depth experienced by the Ca^+, Xe^+, $NH_3{}^+$ and $ND_3{}^+$ ions for the experimental conditions used in this study. Note that the trap depth for both $NH_3{}^+$ (at 7.54 eV) and $ND_3{}^+$ (at 6.32 eV) is significantly greater than the energy released from the charge exchange reaction, resulting in efficient trapping of these product ions. As the kinetic energy of the ammonia ions produced following charge transfer is dissipated within a few ms as a result of efficient sympathetic cooling,[2] we assume that the ammonia ions are trapped with 100% efficiency.

Ion	Trap depth (eV)
Ca^+	2.87
Xe^+	0.46
${NH_3}^+$	7.54
${ND_3}^+$	6.32

Supplementary Table I. Trap depths experienced by the species involved in the charge transfer reactions.

Supplementary Note 3. Comparison between experimental images and MD simulations

Simulated images are produced by a custom-written MD simulation software package. The MD simulations replicate the experimental conditions and model Coulomb crystals in a linear Paul trap. The code calculates the net force F_{total} experienced by each ion

$$F_{\text{total}} = F_{\text{trap}} + F_{\text{Coulomb}} + F_{\text{cooling}} + F_{\text{heating}}, \tag{2}$$

where F_{trap} is the trapping force for the interaction of the ion with the trap fields, F_{Coulomb} the Coulomb force, F_{cooling} a force term related to the cooling mechanism, and F_{heating} a force term that models heating due to photon-recoil or collisions with background gas in the trap. The code integrates Newton's equations of motion to establish the positions of all ions in the crystal. The simulations yield a series of images that can be directly compared with the experimental images.

Through quantitative comparison of the simulated and experimental images using the software package GIMP, one can establish the number of ions of each species within the Coulomb crystal at a given moment in time. The accuracy of this method is ±10 ions for up to several hundred Ca^+ ions in a mono-component Coulomb crystal. For Xe^+ ions—because of their high mass-to-charge ratio and the resulting weaker confining forces imposed on them—the accuracy with which we can establish the number of these species is poor (±50 ions for bi-component crystals with up to several hundred ions). However, the number of initial Xe^+ ions present can be inferred retrospectively from the final number of ammonia ions in the dark core once a reaction is complete. Confirmation that a reaction is complete is verified using ToF-MS data. As ammonia ions have a lower mass-to-charge ratio than the laser-cooled Ca^+ ions, they are located along the trap axis and their number can be accurately established to within ±5 ions.

Supplementary Note 4. Reaction rate constants

The charge exchange reaction between Xe^+ and NH_3 (or ND_3) is a one-to-one process,

$$\frac{\mathrm{d}[\mathrm{Xe}^+]}{\mathrm{d}t} = -\frac{\mathrm{d}[\mathrm{NH}_3^+]}{\mathrm{d}t} = -k_2[\mathrm{Xe}^+][\mathrm{NH}_3], \tag{3}$$

where $[Xe^+]$ and $[NH_3]$ represent the number densities of each reactant species and k_2 is the bimolecular reaction rate constant. Ammonia is introduced effusively into the chamber via a high-precision leak valve. We assume that there is a constant flow of ammonia into the chamber, allowing the rearrangement of Supplemntary Equation (3) into a pseudo-first order equation

$$[\mathrm{NH}_3^+]_t = [\mathrm{Xe}^+]_0 \left(1 - e^{-k_1 t}\right), \tag{4}$$

where k_1 is the pseudo-first order rate constant given by

$$k_1 = k_2[\mathrm{NH}_3]. \tag{5}$$

k_1 is calculated by plotting the number of ${NH_3}^+$ (or ${ND_3}^+$) ions established from the comparison with MD simulations as a function of time. The data points are subsequently fit to an exponential growth curve that follows the equation

$$[\mathrm{NH_3}^+]_t = [\mathrm{Xe}^+]_0(1 - e^{-k_1(t-t_0)}) + [\mathrm{NH_3}^+]_0. \tag{6}$$

The term $[\mathrm{NH_3}^+]_0$ is included in Supplementary Equation (6) to account for any ammonia ions present in the dark core prior to admitting ammonia into the chamber (see Supplementary Note 1). The first few data points recorded for each reaction do not fit well to the exponential growth model of Supplementary Equation (4). This is due to the finite time that the ammonia partial pressure needs to reach the desired value. The t_0 term in Supplementary Equation (6) accounts for the uncertainty in the time that the charge exchange reaction started (*i.e.* to allow for a delay between when the leak valve is opened and when the partial pressure of ammonia reaches the desired stable value in the chamber). To establish when uniform conditions have been achieved, we convert Supplementary Equation (6) to a logarithmic form and fit the data to a linear equation

$$ln(1 - \frac{[\mathrm{NH_3^+}]_t - [\mathrm{NH_3^+}]_0}{[\mathrm{Xe^+}]_0}) = -k_1(t - t_0). \tag{7}$$

Points that clearly deviate from the linear fit of Supplementary Equation (7) at early reaction time are discarded (see Supplementary Figure 1). Note that the linear fit is used only to identify when the partial pressure of ammonia became stable, and not for the calculation of the pseudo-first order rate constants.

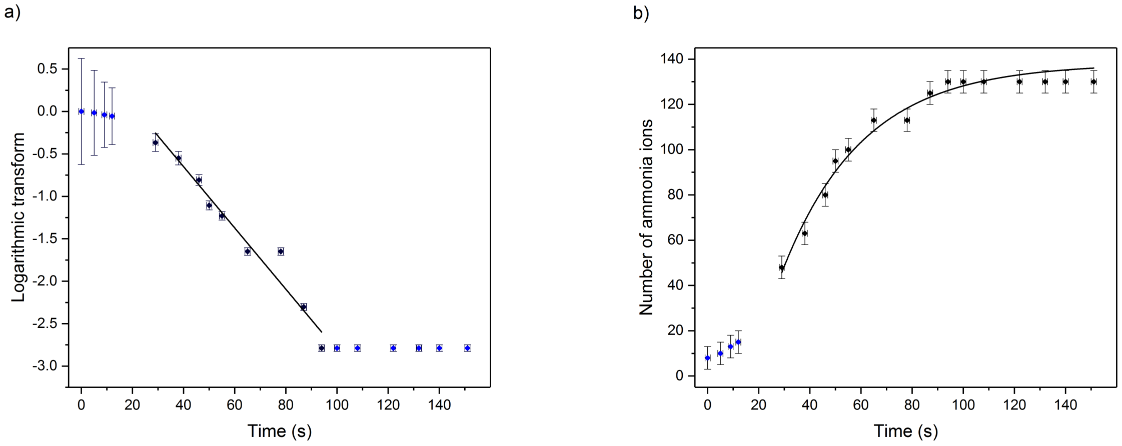

Supplementary Figure 1. Calculation of the experimental reaction rate constants. (a) Logarithmic form of Supplementary Equation (7). The linear fit is excellent once the first four points are discarded. The later points (appearing as a horizontal line) deviate from the earlier linear trend, as the reaction has gone to completion and so the number of ammonia ions is no longer changing. (b) Exponential growth fit to Supplementary Equation (6), taking into account the data points recorded once conditions were stable. Error bars of $E_y = \pm 5$ ions and $E_x = \pm 1$ second account for the accuracy of the MD simulations and experimental uncertainty in the reaction time, respectively.

To calculate bimolecular rate constants (k_2) from the pseudo-first order rate constants (k_1), one can use the equation

$$k_2 = \frac{k_1}{[\mathrm{NH_3}]} = \frac{k_1}{N/V} = \frac{k_1 k_\mathrm{B} T}{P}, \tag{8}$$

where k_B is the Boltzmann constant, T is the temperature of the ammonia molecules (290 K in our laboratory) and P is the partial pressure of ammonia in the trap chamber (3×10^{-9} mbar). Supplementary Table II summarises the calculated pseudo-first order and bimolecular reaction rate constants for the $\mathrm{Xe^+} + \mathrm{NH_3}$ and $\mathrm{Xe^+} + \mathrm{ND_3}$ systems. The equation used to calculate the uncertainty range of the bimolecular rate constant, k_2, is established from the propagation of the uncertainty associated with each of the terms of Supplementary Equation 8,

$$\sigma_{k_2} = k_2\sqrt{(\frac{\sigma_{k_1}}{k_1})^2 + (\frac{\sigma_P}{P})^2}. \tag{9}$$

Isotopologue	k_1 (s^{-1})	k_2 (cm^3 s^{-1})
NH_3	$3.3(2) \times 10^{-2}$	$0.36(2) \times 10^{-9}$
ND_3	$11(2) \times 10^{-2}$	$1.2(2) \times 10^{-9}$

Supplementary Table II. Pseudo-first order and bimolecular reaction rate constants for the Xe^+ + NH_3 and Xe^+ + ND_3 systems.

Supplementary Note 5. ADO rate constants

We compare the calculated reaction rate constants with the average dipole orientation (ADO) theory for ion-polar neutral reactions.[3] To calculate the ADO rate constants in SI units, we use the equation

$$k_{\mathrm{ADO}} = Q\sqrt{\frac{\pi\alpha}{\varepsilon_0\mu}} + \frac{Qc\mu_D}{\varepsilon_0}\sqrt{\frac{1}{2\pi\mu k_B T}}, \tag{10}$$

where Q is the charge of the ion, α the polarisability of the neutral molecule, ε_0 the permittivity of free space, μ the reduced mass of the ion-neutral system, μ_{D} the dipole moment of the neutral, k_{B} the Boltzmann constant and T the temperature of the reaction. The parameter c accounts for the average orientation of the dipole of the neutral species during the reaction and depends on the temperature of the system and the ratio $\frac{\mu_{\mathrm{D}}}{\alpha^{1/2}}$.[4] The first term of Supplementary Equation (10) is the Langevin reaction rate constant. Supplementary Table III shows the polarisability and dipole moment of ammonia, the parameter c and the calculated ADO rate constants for our experimental conditions.

Isotopologue	α (cm^3)[5,6]	μ_{D} (D)[6,7]	c	k_{ADO} (cm^3 s^{-1})
NH_3	2.16×10^{-24}	1.47	0.227	1.8×10^{-9}
ND_3	1.88×10^{-24}	1.50	0.232	1.6×10^{-9}

Supplementary Table III. Parameters used to calculate the ADO rate constants for comparison with our experimental rates.

Supplementary References

[1] D. J. Levandier and Y.-H. Chiu. A guided-ion beam study of the reactions of Xe^+ and Xe^{2+} with NH_3 at hyperthermal collision energies. *J. Chem. Phys.*, **133**, 154304 (2010).

[2] S. Schiller and C. Lämmerzahl. Molecular dynamics simulation of sympathetic crystallization of molecular ions. *Phys. Rev. A*, **68**, 053406 (2003).

[3] T. Su and M. T. Bowers. Theory of ion-polar molecule collisions. Comparison with experimental charge transfer reactions of rare gas ions to geometric isomers of difluorobenzene and dichloroethylene. *J. Chem. Phys.*, **58**, 3027–3037 (1973).

[4] T. Su and M. T. Bowers. Parameterization of the average dipole orientation theory: temperature dependence. *Int. J. Mass Spectrom. Ion Process.*, **17**, 211–212 (1975).

[5] R. L. Woodin and J. L. Beauchamp. Binding of Li^+ to Lewis bases in the gas phase. Reversals in methyl substituent effects for different reference acids. *J. Am. Chem. Soc.*, **100**, 501–508 (1978).

[6] E. A. Halevi, E. N. Haran and B. Ravid. Dipole moment and polarizability differences between NH_3 and ND_3. *Chem. Phys. Lett.*, **1**, 475–476 (1967).

[7] J. M. Hollas. *Modern Spectroscopy*, John Wiley & Sons, UK (2004).
